# Genomic Prediction of Two Complex Orthopedic Traits Across Multiple Pure and Mixed Breed Dogs

**DOI:** 10.3389/fgene.2021.666740

**Published:** 2021-09-22

**Authors:** Liping Jiang, Zhuo Li, Jessica J. Hayward, Kei Hayashi, Ursula Krotscheck, Rory J. Todhunter, You Tang, Meng Huang

**Affiliations:** ^1^ College of Mathematics, Jilin University, Changchun, China; ^2^ Electrical and Information Engineering College, Jilin Agricultural Science and Technology University, Jilin, China; ^3^ Department of Biomedical Sciences, College of Veterinary Medicine, Cornell University, Ithaca, NY, United States; ^4^ Department of Clinical Sciences and Cornell Veterinary Biobank, College of Veterinary Medicine, Cornell University, Ithaca, NY, United States; ^5^ Institute for Behavioral Genetics, University of Colorado Boulder, Boulder, CO, United States

**Keywords:** canine, hip and elbow dysplasia, rupture of the cranial cruciate ligament, genomic prediction, across breeds

## Abstract

Canine hip dysplasia (CHD) and rupture of the cranial cruciate ligament (RCCL) are two complex inherited orthopedic traits of dogs. These two traits may occur concurrently in the same dog. Genomic prediction of these two diseases would benefit veterinary medicine, the dog’s owner, and dog breeders because of their high prevalence, and because both traits result in painful debilitating osteoarthritis in affected joints. In this study, 842 unique dogs from 6 breeds with hip and stifle phenotypes were genotyped on a customized Illumina high density 183 k single nucleotide polymorphism (SNP) array and also analyzed using an imputed dataset of 20,487,155 SNPs. To implement genomic prediction, two different statistical methods were employed: Genomic Best Linear Unbiased Prediction (GBLUP) and a Bayesian method called BayesC. The cross-validation results showed that the two methods gave similar prediction accuracy (*r* = 0.3–0.4) for CHD (measured as Norberg angle) and RCCL in the multi-breed population. For CHD, the average correlation of the AUC was 0.71 (BayesC) and 0.70 (GBLUP), which is a medium level of prediction accuracy and consistent with Pearson correlation results. For RCCL, the correlation of the AUC was slightly higher. The prediction accuracy of GBLUP from the imputed genotype data was similar to the accuracy from DNA array data. We demonstrated that the genomic prediction of CHD and RCCL with DNA array genotype data is feasible in a multiple breed population if there is a genetic connection, such as breed, between the reference population and the validation population. Albeit these traits have heritability of about one-third, higher accuracy is needed to implement in a natural population and predicting a complex phenotype will require much larger number of dogs within a breed and across breeds. It is possible that with higher accuracy, genomic prediction of these orthopedic traits could be implemented in a clinical setting for early diagnosis and treatment, and the selection of dogs for breeding. These results need continuous improvement in model prediction through ongoing genotyping and data sharing. When genomic prediction indicates that a dog is susceptible to one of these orthopedic traits, it should be accompanied by clinical and radiographic screening at an acceptable age with appropriate follow-up.

## Introduction

Canine hip dysplasia (CHD) is a common complex trait that results in joint instability and painful osteoarthritis (OA). The estimated heritability of CHD ranges from 0.2 to 0.6 (Breur and Lambrecht, 2012; Oberbauer et al., 2017). Radiographic imaging can help make a diagnosis but is imperfect when dogs are immature (Smith et al., 1998; Ginja et al., 2010). The Norberg angle, a quantitative measure of hip congruity, is correlated with the traditional hip score accorded by the Orthopedic Foundation for Animals (OFA) (https://www.ofa.org), but in and of itself is not a perfect predictor based on laxity measures of CHD (Gaspar et al., 2016). Other measurements of hip laxity and subluxation have improved diagnostic capability (Lust et al., 2001; Todhunter et al., 2003), but the phenotype is not an accurate predictor of genotype for complex traits. Even after 60 years of controlled breeding in Sweden, a recent study suggests that further improvement in hip conformation is likely to rely on estimated breeding values and genomic selection (Hedhammar, 2020). Even when estimated breeding values for hip conformation are applied in closed colonies like the Seeing Eye Foundation, although the prevalence and trait severity decrease over visual observation of pedigrees for breeding decisions, CHD still occurs, leading the authors to suggest that genomic approaches are needed for maximum impact on trait severity and prevalence (Leighton et al., 2019).

Rupture of the cranial cruciate ligament (RCCL) is the most common cause of pelvic limb lameness in dogs and, like CHD, induces the osteoarthritic cascade. The reported heritability of RCCL ranges from 0.15 to 0.27 based on pedigree (Wilke et al., 2006) and up to 0.88 based on single nucleotide polymorphism estimates (Cook et al., 2020).

Estimated breeding values can help to improve the genetic and phenotypic quality of a closed population where breeding can be controlled (Leighton et al., 2019). Genetic marker information can be used in the calculation of genomic breeding values which can be related to the dogs’ estimated breeding values. The reference population, therefore, has to be genotyped. Then the genomic information can be used to predict the genetic merit of new offspring based on the reference population (Contaldi et al., 2021). This method can be applied in closed populations where breeding can be controlled, as in experimental breeding colonies (Zhang et al., 2009), and in-service dog organizations.

However, estimated breeding values are often not available to the general public. The genomic prediction could be employed to assist purchase and breeding decisions when accurate deep pedigree information and accompanying phenotypic data are not available. Such prediction technologies could assist in the clinical diagnosis of these complex orthopedic traits, especially in puppies when therapeutic intervention has windows of opportunity and imaging methods are imprecise. Previous empirical studies have indicated that the genomic prediction of CHD is feasible in a single purebred population (Sánchez-Molano et al., 2015) and a limited number of multiple breeds (Guo et al., 2012). However, there are several limitations to the implementation of genomic prediction including sample size, multiple susceptible breeds, unknown genetic relationships, and the increased difficulty in predicting a complex trait phenotype in which environmental (non-genetic) inputs play a large role. The collection of multiple breeds across populations could expand the sample size. The genetic relatedness among genotyped individuals may affect the accuracy of prediction, especially when models based on a genetic relationship matrix, such as the mixed linear model, are used for prediction (Scutari et al., 2016).

In this paper, we investigate the accuracy of genomic prediction using Genomic Best Linear Unbiased Prediction (GBLUP) and BayesC in DNA array data and imputed data to predict two common, and clinically important, complex orthopedic traits. The impact of associated markers on the prediction accuracy was also assessed. The experimental dataset included 6 pure breeds of dogs. The accuracy of each model is evaluated by cross-validation. We measure the prediction accuracy with Pearson correlation and area under the receiver operator characteristic curve (AUC), as we have both a quantitative trait (CHD) and a qualitative trait (RCCL) in this study.

## Materials and Methods

### Genotype Data

**FIGURE 1:**
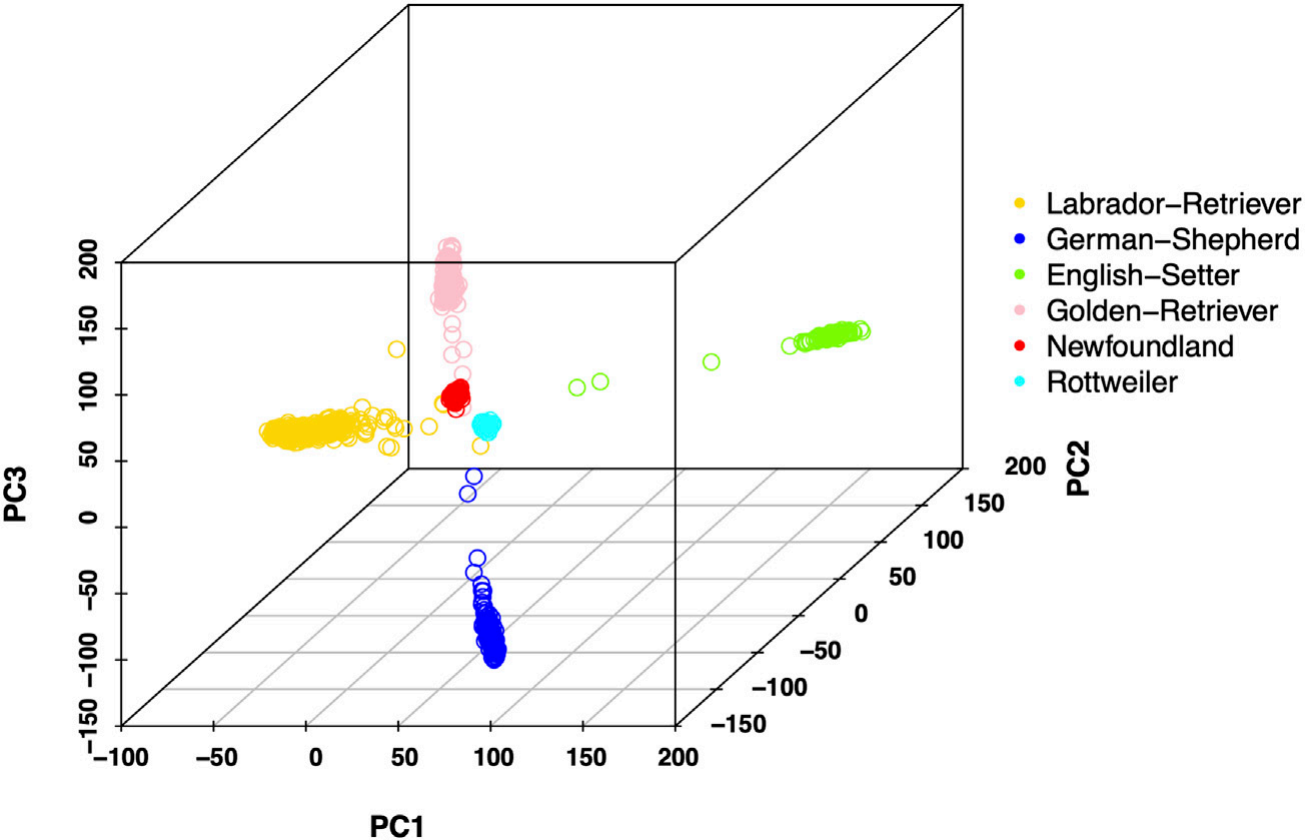
Principal component (PC) plot of all samples. The first 3 PCs are used to show the genetic relationship of different breeds. All breeds are marked by a different color.

Publicly available 183 k semi-custom CanineHD array genotype dataset was used in this study (Hayward et al., 2016). For DNA array data, 160,470 SNPs without missing genotypes were used in the analysis. Principal component analysis (PCA) of the genotypes was conducted using PLINK 2.0 (Chang et al., 2015), and the first 3 PCs were used to draw PCA plots (Figure 1). We also test the prediction accuracy of associated markers, which were the SNPs located in the region of the 147 reported possible causal genes with 10 kb extension of both the upstream and downstream boundary (Zhou et al., 2010; Pfahler and Distl, 2012; Fels et al., 2014; Lavrijsen et al., 2014; Bartolomé et al., 2015; Huang et al., 2017; Hatzikotoulas et al., 2018; Mikkola et al., 2019; Todhunter et al., 2019; Zamborsky et al., 2019; Kang et al., 2020; Mikkola et al., 2021). A total of 808 SNPs located within the boundary of these 147 possible causal genes associated with CHD or RCCL were selected as associated markers from DNA array data. From the imputed genotype data, 54,858 SNPs were selected as associated markers.

We also use the imputed genotype data from Hayward et al., 2019 to evaluate the prediction performance of GBLUP. These imputed data were created using a reference set of 365 canine whole genome sequences, which were then imputed across phased DNA array genotype data (Hayward et al., 2019). Accuracy for this imputation panel was calculated to be 88.4% overall, and 89.7% in the purebred dogs only (Hayward et al., 2019). For imputed genotype data, 20,487,155 markers without missing genotypes were used for genomic prediction.

### Phenotype Data

We selected 842 dogs from 6 breeds for genomic prediction, which included 97 German Shepherd dogs, 80 English Setters, 137 Golden Retrievers, 398 Labrador Retrievers, 68 Newfoundlands, and 62 Rottweilers. All the dogs with orthopedic phenotypes were examined at Cornell University and all from the northeastern United States. Supplementary Figure S1 shows the distribution of the observed phenotypes. One hundred and eight dogs had CHD, RCCL, and weight data, and 406 dogs had any two of these three phenotypes. For CHD, the Norberg angle (NA) of both hips was measured radiographically in 608 dogs. The NA ranges from less than 50°–120°. The average NA over both hips was used as the CHD phenotype. Norberg angles below 75° were truncated to 75° to approximate a normal distribution. Rupture of the cranial cruciate ligament was diagnosed by palpation and/or radiography, and 199 cases and 257 controls were included in the analysis. Body weight was recorded for 292 dogs.

### BayesC Model

For the BayesC model, sex and breed were fitted as the fixed effects and the genotypes were fitted as the random effect. The prediction analysis of BayesC was implemented using the R package BGLR (Pérez and de los Campos, 2014). The model equation is written as

y=η+e

Where *y* is a (n×1) vector of observed phenotypes, η is a (n×1) vector of linear predictors, *e* is a (n×1) vector of the independent normal model residuals that $e\;∼\;N\left(0,\;I\sigma_{e}^{2}\right),$ where I is the identity matrix. The linear predictor η is given as follows:

$$\eta =\mu +X\beta +\overset{L}{\underset{j=1}{\sum }}m_{j}\alpha_{j}$$

where μ is a (n×1) vector of overall mean, and β is (K×1 vector of fixed effect, X is the (n×K) incidence matrix for β,$\;\alpha =\left(\alpha_{1},\cdots ,\alpha_{L}\right)\prime$ is a vector of marker effects (random effect), $\alpha_{j}$ is *jth* marker effect. $m=\left(m_{1},\cdots ,m_{L}\right)$ is the (n×L) genotype matrix, and $m_{j}$ is the *jth* column of m. For BayesC (Habier et al., 2011), the prior distribution of marker effect $\alpha_{j}$ is assigned an independent and identically distributed Gaussian mixture prior, which is a mixture of a point of mass at zero with probability 1−π and normal distribution with probability π; the hyper-parameters of the prior densities and other parameters are the same as the default setting in the BGLR package.

### GBLUP Model

GBLUP is a mixed linear model, which was implemented using the method “RKHS” in the BGLR package (Henderson, 1976; Wahba, 1990). Sex and breed were fitted as the fixed effects, the genotypes were fitted as the random effect. The model is usually written as

y=μ+Xβ+Zg+e  

where *y* is a (n×1) vector of the observed phenotype, μ is the (n×1) vector of overall mean, β is a (K×1) vector of the fixed effects, g is a (n×1) vector of the genetic random effects with $g\;∼\;N\left(0,\;K\sigma_{g}^{2}\right)$, *X* and *Z* are the incidence matrices for β and g, and e is a (n×1) vector of residuals with $e\;∼\;N\left(0,\;I\sigma_{e}^{2}\right)$. *K* is a kinship matrix that was derived from the observed genotype matrix, and I is the identity matrix.

### Simulation

The DNA array genotype data were used to produce simulated phenotypes in six breeds: Labrador Retriever, German Shepherd dog, English Setter, Golden Retriever, Newfoundland, and Rottweiler. The simulated phenotype values included additive genetic effect and residual effect, and both followed the normal distribution. For all six breeds, the simulated phenotypes were controlled by 20 quantitative trait nucleotides QTNs that were randomly selected from all the markers. The heritability in different breeds was randomly sampled from 0.2 to 0.8.

### Evaluation of Prediction Accuracy

**FIGURE 2:**
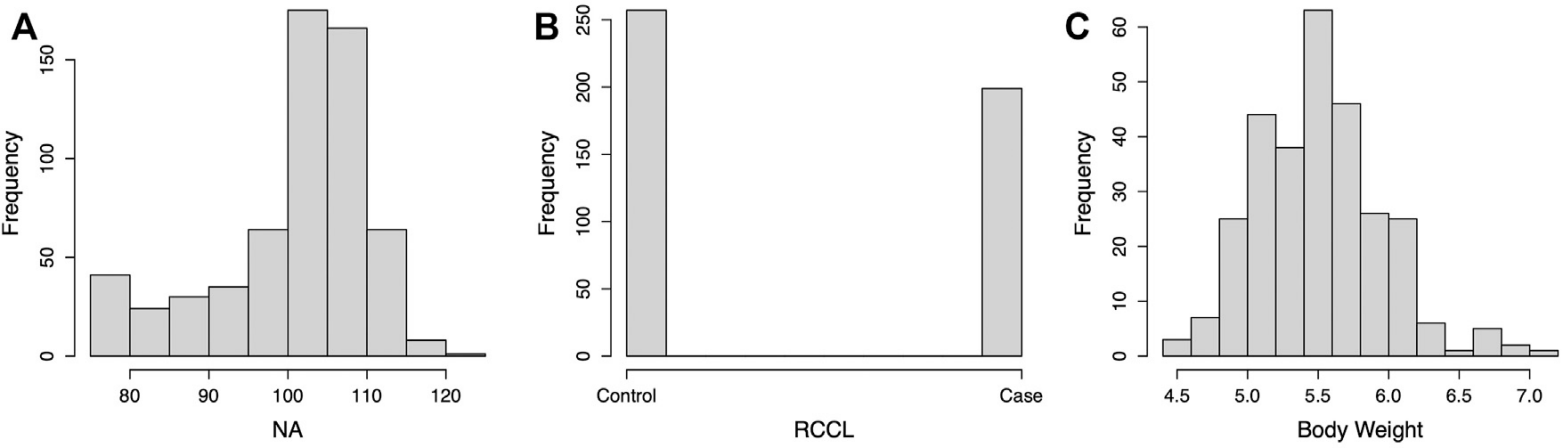
Histogram of the phenotype distribution of canine hip dysplasia (CHD), rupture of cranial cruciate ligament (RCCL), and body weight. NA = Norberg angle.

The five-fold cross-validation was applied to assess the performance of each model and the feasibility of genomic prediction of the current traits in this population (Figure 2). One hundred rounds of cross-validation were conducted for each model. In each round, the population was divided into five subgroups. Each subgroup was considered as the validation panel, and the remaining four subgroups were the reference panel. The 5-fold cross-validation method was employed to estimate the prediction accuracy. Two sampling strategies were used in this study: random sampling and single breed sampling. For random sampling, the population was randomly divided into five subgroups. For single breed sampling, only dogs from the breed with the highest sample size (Labrador Retriever) were randomly divided into five subgroups, the other dogs from other breeds were always grouped as the reference panel when each subgroup was considered as the validation panel.

The Pearson correlation was calculated between the genomic prediction results and the observed phenotype in validation populations. The mean correlation results in each 5-fold cross-validation round were used to obtain the final mean and standard deviation of all rounds of cross-validation for each trait and model. The AUC (the area under the receiver operator characteristic curve) was also employed to evaluate the accuracy using the normalized prediction results and binary phenotype value (NA was converted to a binary value with a cutoff set to 105°). The R package pROC (Turck et al., 2011) was used to calculate the AUC.

## Results

### Cross-Validation With DNA Array Genotype Data

For CHD, cross-validation results of the average NA showed that both models had similar performance, with the randomly sampled training panel from all six breeds. The average Pearson correlation coefficients was 0.355 (BayesC) and 0.359 (GBLUP). The average correlation of the AUC results were 0.705 (BayesC) and 0.703 (GBLUP), which is a medium level of the prediction accuracy and consistent with Pearson correlation results (Table 1, Supplementary Table S1). We also implemented the cross-validation in a single breed (Labrador Retriever) and found that both models had similar performance (Supplementary Table S1). The average Pearson correlation coefficients dropped to 0.294 (BayesC) and 0.293 (GBLUP). To test whether more dogs from other breeds could enhance the prediction accuracy, we added all the other dogs into the reference population of the Labrador Retriever cross-validation. The addition of dogs from other breeds did not enhance the prediction accuracy in CHD (Supplementary Table S1). For RCCL, BayesC and GBLUP resulted in slightly higher Pearson correlation coefficients and AUC when compared to CHD (Table 1). However, the results from single breed cross-validation have a higher accuracy (0.54 in Pearson correlation and 0.80 in AUC) than the whole population cross-validation (Supplementary Table S1). More reference dogs from other breeds did not enhance the prediction accuracy. Body weight was used as a positive control in cross-validation (random sampling). The average accuracy results of the Pearson correlation in random sampling were 0.525 (BayesC) and 0.509 (GBLUP), which dropped to 0.311 (BayesC) and 0.304 (GBLUP) using the single-breed sampling approach (Supplementary Table S1).

**TABLE 1:** The cross-validation results (averaged Pearson correlation) based on random sampling for canine hip dysplasia (CHD), rupture of the cranial cruciate ligament (RCCL), and body weight. NA = Norberg angle, GBLUP = Genomic Best Linear Unbiased Prediction Model. ALL = using all DNA array markers in multi-breed population, ASS-ALL = using associated markers in multi-breed population, ASS-GR = using associated markers in Golden Retriever dogs, ASS-LR = using associated markers in Labrador Retriever dogs, RAN-ALL = using randomly selected markers in multi-breed population, RAN-GR = using randomly selected markers in Golden Retriever dogs, RAN-LR = using randomly selected markers in Labrador Retriever dogs, IMP = using all the imputed markers in multi-breed population.

Trait	Model	ALL	ASS-ALL	ASS-GR	ASS-LR	RAN-ALL	RAN-GR	RAN-LR	IMP
NA	BayesC	0.355	0.289	0.159	0.229	0.300	0.376	0.185	—
	GBLUP	0.359	0.285	0.159	0.226	0.304	0.379	0.179	0.366
RCCL	BayesC	0.377	0.257	0.043	0.328	0.308	0.017	0.402	—
	GBLUP	0.383	0.249	0.049	0.326	0.301	0.024	0.411	0.366
Body Weight	BayesC	0.525	0.510	0.027	0.211	0.486	0.095	0.190	—
	GBLUP	0.509	0.496	0.007	0.211	0.483	0.14	0.193	0.521

### Cross-Validation With Different Marker Density

To explore the impact of marker density in the prediction accuracy, we evaluated the accuracy of genomic prediction using imputed genotype data (high-density data) and associated SNPs (low-density data) in both orthopedic traits and body weight. For imputed genotype data, we only tested the performance of GBLUP as the computing cost of the BayesC method is too high. Using 808 associated SNPs from the possible causal genes that were selected from the published GWAS reports led to a reduction of prediction accuracy relative to the accuracy using all the DNA array data in all three traits. When using 808 SNPs randomly selected in a multi-breed population, both CHD and RCCL had an even higher accuracy, which concords with previous study in CHD with Labrador Retrievers (Sánchez-Molano et al., 2015). However, using the single breed approach, the accuracy of CHD with randomly selected SNPs in Golden Retrievers was increased, while the accuracy in Labrador Retrievers was decreased, relative to the accuracy with associated SNPs. For RCCL, the results were the opposite (Table 1, Supplementary Tables S2–S4). These results imply that the associated SNPs may have different performances in different breeds. The results from imputation genotype data had the same trend and limited success in increasing the prediction accuracy (Table 1, Supplementary Tables S5–S7).

### Simulation

To further validate the results above, we simulated a continuous phenotype in 6 breeds. We explored the prediction accuracy of the cross-validation within one breed (Labrador Retriever), and the whole population (all 6 breeds). For the single breed validation, the other five breeds were also added to the reference population to test if they affected the prediction accuracy. The results showed that random sampling in a multi-breed population had higher accuracy than a single breed population. More dogs from other breeds in the reference population did not increase the prediction accuracy (Supplementary Table S2).

## Discussion

This study evaluated the performance of genomic prediction in two complex orthopedic traits using GBLUP and BayesC methods, which gave similar prediction accuracy results. Using published associated markers, we expected to see an increase in the prediction accuracy. However, the accuracy of the prediction with the associated SNPs was less than the prediction with all 160 k informative DNA array markers. The randomly selected 808 SNPs had a similar prediction accuracy to the associated markers, which is consistent with a previous report (Sánchez-Molano et al., 2015). The higher marker density (imputed genotype data) also did not improve the prediction accuracy. By increasing the training panel size, the prediction accuracy was improved, but more individuals from other breeds in the training panel did not enhance the prediction accuracy.

In the genomic prediction of complex traits, the genetic effect of a large number of markers was evaluated and used to estimate breeding value. The GBLUP method assumes that each marker has a genetic effect on the target trait, while the BayesC approach assumes that only some markers have a genetic effect on the trait. The results in this study indicated that the average prediction accuracy from cross-validation was not significantly different between GBLUP and BayesC in CHD and RCCL, which is consistent with previous studies (Zhu et al., 2012; Sánchez-Molano et al., 2014; Sánchez-Molano et al., 2015; Baker et al., 2020).

Theoretically, increasing the density of markers could fill any gaps which might increase linkage disequilibrium (LD) between the testing markers and any quantitative trait locus (QTL), thus enhancing the prediction accuracy. Although the imputed genotype data had >120-fold more markers than the DNA array data, the improvement of accuracy was limited in both traits as tested using the GBLUP model. The results indicated that the higher marker density, without any selection, did not increase the accuracy of genomic prediction. For GBLUP, changing the marker density could only affect the precision of the genetic relationship matrix as long as the marker density was not high enough initially. This suggests that these 160 k markers from the DNA array cover most of the QTLs. Another possible reason is that the increase in the accuracy may be offset by the introduced imputation error. A large number of markers from genotyping or imputation could include some level of redundant markers without a genetic effect. Thus, selected markers could give a similar prediction accuracy in contrast with the full marker dataset. The selection of causal SNPs, or a weighted kinship, could enhance the accuracy of prediction (Zhang et al., 2010; Yin et al., 2020), which is consistent with our prediction results for body weight, but not for CHD and RCCL. The results of CHD and RCCL showed that the markers reported as associated with these traits gave lower accuracy than randomly chosen markers in both the DNA array dataset and imputation dataset. This result is consistent with the previous report in CHD (Sánchez-Molano et al., 2015). The potential reason is that only a small number of genes with larger genetic effects on CHD and RCCL had been detected thus far. Consequently, randomly selected markers could cover more discrete genome regions, which contain QTL with small genetic effects.

Nonetheless, previous simulation studies showed that the accuracy of prediction is sensitive to sample size, but not marker density (Iheshiulor et al., 2016). Generally, the sample size of the reference panel and the genetic relationship between reference and validation panels are two key factors for the accuracy of genomic prediction. The larger reference population could give higher prediction accuracy. However, it is hard to collect enough dogs that were affected with CHD or RCCL within a single breed and originating in a single center. Combining multi-breeds into a common reference population has been used in cattle breeding (VanRaden et al., 2009; Lund et al., 2011; Chen et al., 2015; Rolf et al., 2015; Song et al., 2019). It should be noted that the population used for cross-validation included multiple breeds. Although the multi-breed population has a more complex genetic background than a single breed population, we could gain higher prediction accuracy by increasing the sample size, because increasing the size of the phenotype dataset could produce higher power to distinguish genetic effects from random noise (Iheshiulor et al., 2016). The single breed cross-validation for CHD, the prediction accuracy in a single breed is much lower than the accuracy in a multi-breed population. However, for RCCL, the accuracy from a single breed is slightly higher than the results from the multi-breed population. We also added the dogs from the other breeds to the reference population to test if this would affect the prediction accuracy. The results showed that the addition of more dogs from other breeds did not increase the prediction accuracy (Supplementary Table S1). Additionally, we simulated quantitative phenotypes using DNA array genotype data in six breeds (German Shepherd dog, English Setter, Golden Retriever, Labrador Retriever, Newfoundland, Rottweiler). For different breeds, the heritability was varied, and the locations of simulated causal SNPs were the same. The trend of the prediction accuracy is consistent with the results of the real phenotype in the single breed population and the multi-breed population.

To ensure the predictions with multi-models were correctly implemented, we also predicted body weight using GBLUP and BayesC, with different marker densities. The prediction accuracy of associated markers was very similar to the accuracy using the full set of DNA array data and higher than the accuracy of randomly-selected markers. This result is anticipated as most of the associated genes are also associated with growth and development and approximately 80–88% of the phenotypic variance of a purebred dog’s body weight and height can be estimated from 17 QTL (Hayward et al., 2016).

A limitation to genomic prediction accuracy is that an unknown subset of the control population for rupture of the cranial cruciate ligament might eventually succumb to the trait. Most dogs develop cranial cruciate ligament disease at the age of 4–10 years of age with a median age of 5.1 years (Powers et al., 2005). Important factors associated with RCCL are the dog’s sex, whether or not the dog is neutered, and its body weight. An ideal study would be to add body weight, sex (male, female, or neutered male or female) and age at diagnosis to the model. Because RCCL is a polygenic trait with an unknown number of mutations contributing to the trait, we do not know what overall effect modeling these fixed effects as covariates in the linear model would have on the additive effect of the markers. For CHD measured as the NA, there would be no effect of age on this trait, because the NA is established at skeletal maturity (8 months of age in large breed dogs) (Powers et al., 2005). Body weight and age will affect the progression of secondary osteoarthritis that results from CHD but we are not mapping secondary osteoarthritis here.

## Conclusion

This study indicates that the genomic prediction of both complex canine orthopedic traits is feasible in a multi-breed population if the dogs in the reference population and the validation population came from the same breed. Dogs from other breeds in the reference population do not increase the accuracy. The performance of multiple prediction models shows that there is a small difference in prediction accuracy between different orthopedic traits for different models. A higher marker density does not increase the prediction accuracy, and a lower marker density could decrease the accuracy. Further, using associated markers did not improve prediction accuracy in CHD and RCCL. With more genetically related individuals in the reference population, especially when the population size is limited, the accuracy of genomic prediction could be improved.

## Data Availability

The original contributions presented in the study are included in the article/Supplementary Material, further inquiries can be directed to the corresponding authors.
