# Supplementary material for: Genomic Prediction of Two Complex Orthopedic Traits Across Multiple Pure and Mixed Breed Dogs
Source: Front Genet. 2021 Sep 22;12:666740. doi: 10.3389/fgene.2021.666740 (PMC8492927; doi:10.3389/fgene.2021.666740)

SUPPLEMENTAL TABLES

Table S1. The cross-validation results based on DNA array data for CHD, RCCL, and body weight. CHD = canine hip dysplasia, RCCL = rupture of the cranial cruciate ligament, NA = Norberg angle, AUC=Area Under the Receiver Operator Characteristic Curve, GBLUP = Genomic Best Linear Unbiased Prediction, M1 = random sampling in multi-breed population, M2 = random sampling in Labrador Retriever dogs and adding dogs from other breeds in the reference population, S1 = random sampling in Labrador Retriever dogs with fixed effect (sex).

|  | Trait | Model | Pearson Correlation | | AUC | |
| --- | --- | --- | --- | --- | --- | --- |
|  |  |  | Average | SD | Average | SD |
| M1 | NA | BayesC | 0.355 | 0.015 | 0.705 | 0.008 |
|  |  | GBLUP | 0.359 | 0.018 | 0.703 | 0.010 |
|  | RCCL | BayesC | 0.377 | 0.029 | 0.725 | 0.010 |
|  |  | GBLUP | 0.383 | 0.032 | 0.728 | 0.012 |
|  | Body Weight | BayesC | 0.525 | 0.058 | ---- | ---- |
|  |  | GBLUP | 0.509 | 0.055 | ---- | ---- |
| M2 | NA | BayesC | 0.297 | 0.027 | 0.694 | 0.017 |
|  |  | GBLUP | 0.301 | 0.030 | 0.684 | 0.017 |
|  | RCCL | BayesC | 0.479 | 0.018 | 0.790 | 0.012 |
|  |  | GBLUP | 0.480 | 0.019 | 0.789 | 0.013 |
|  | Body Weight | BayesC | 0.311 | 0.038 | ---- | ---- |
|  |  | GBLUP | 0.304 | 0.033 | ---- | ---- |
| S1 | NA | Bayesian C | 0.280 | 0.035 | 0.692 | 0.019 |
|  |  | GBLUP | 0.274 | 0.034 | 0.692 | 0.019 |
|  | RCCL | Bayesian C | 0.534 | 0.019 | 0.799 | 0.015 |
|  |  | GBLUP | 0.533 | 0.021 | 0.798 | 0.016 |

Table S2. The cross-validation results for simulated phenotype based on DNA array data. NA = Norberg angle, AUC=Area Under the Receiver Operator Characteristic Curve, GBLUP = Genomic Best Linear Unbiased Prediction Model, SP1 = random sampling in multi-breed population, SP2 = random sampling in Labrador Retriever dogs, SP3 = random sampling in Labrador Retriever dogs with dogs from other breeds in the reference population.

| Trait | Model | Pearson Correlation | |
| --- | --- | --- | --- |
|  |  | Average | SD |
| SP1 | BayesC | 0.380 | 0.009 |
|  | GBLUP | 0.377 | 0.010 |
| SP2 | BayesC | 0.180 | 0.024 |
|  | GBLUP | 0.188 | 0.022 |
| SP3 | BayesC | 0.167 | 0.020 |
|  | GBLUP | 0.167 | 0.022 |

Table S3. The cross-validation results, using BayesC and GBLUP model, based on 808 associated SNPs from DNA array data for canine hip dysplasia (CHD), rupture of the cranial cruciate ligament (RCCL), and body weight. NA = Norberg angle, AUC=Area Under the Receiver Operator Characteristic Curve. M1 = random sampling in multi-breed population, M2 = random sampling in Golden Retriever dogs, M3 = random sampling in Labrador Retriever dogs, M4 = random sampling in Labrador Retriever dogs and adding dogs from other breeds in the reference population.

|  | Trait | Model | Pearson Correlation | | AUC | |
| --- | --- | --- | --- | --- | --- | --- |
|  |  |  | Average | SD | Average | SD |
| M1 | NA | BayesC | 0.289 | 0.018 | 0.677 | 0.008 |
|  |  | GBLUP | 0.285 | 0.018 | 0.675 | 0.008 |
|  | RCCL | BayesC | 0.257 | 0.035 | 0.655 | 0.016 |
|  |  | GBLUP | 0.249 | 0.037 | 0.651 | 0.016 |
|  | Body Weight | BayesC | 0.510 | 0.059 | ---- | ---- |
|  |  | GBLUP | 0.496 | 0.060 | ---- | ---- |
| M2 | NA | BayesC | 0.159 | 0.062 | 0.619 | 0.041 |
|  |  | GBLUP | 0.159 | 0.067 | 0.618 | 0.040 |
|  | RCCL | BayesC | 0.043 | 0.098 | 0.621 | 0.048 |
|  |  | GBLUP | 0.049 | 0.092 | 0.617 | 0.049 |
|  | Body Weight | BayesC | 0.024 | 0.108 | ---- | ---- |
|  |  | GBLUP | 0.007 | 0.109 | ---- | ---- |
| M3 | NA | BayesC | 0.229 | 0.039 | 0.645 | 0.024 |
|  |  | GBLUP | 0.226 | 0.040 | 0.643 | 0.025 |
|  | RCCL | BayesC | 0.328 | 0.027 | 0.690 | 0.017 |
|  |  | GBLUP | 0.326 | 0.027 | 0.688 | 0.017 |
|  | Body Weight | BayesC | 0.211 | 0.041 | ---- | ---- |
|  |  | GBLUP | 0.211 | 0.042 | ---- | ---- |
| M4 | NA | BayesC | 0.234 | 0.027 | 0.630 | 0.018 |
|  |  | GBLUP | 0.227 | 0.025 | 0.625 | 0.017 |
|  | RCCL | BayesC | 0.321 | 0.024 | 0.692 | 0.017 |
|  |  | GBLUP | 0.314 | 0.025 | 0.687 | 0.017 |
|  | Body Weight | BayesC | 0.222 | 0.035 | ---- | ---- |
|  |  | GBLUP | 0.223 | 0.036 | ---- | ---- |

Table S4. The cross-validation results, using BayesC and GBLUP model, based on 808 randomly selected SNPs from DNA array data for canine hip dysplasia (CHD), rupture of the cranial cruciate ligament (RCCL), and body weight. NA = Norberg angle, AUC=Area Under the Receiver Operator Characteristic Curve. M1 = random sampling in multi-breed population, M2 = random sampling in Golden Retriever dogs, M3 = random sampling in Labrador Retriever dogs, M4 = random sampling in Labrador Retriever dogs and adding dogs from other breeds in the reference population.

|  | Trait | Model | Pearson Correlation | | AUC | |
| --- | --- | --- | --- | --- | --- | --- |
|  |  |  | Average | SD | Average | SD |
| M1 | NA | BayesC | 0.300 | 0.016 | 0.675 | 0.008 |
|  |  | GBLUP | 0.304 | 0.015 | 0.674 | 0.008 |
|  | RCCL | BayesC | 0.308 | 0.027 | 0.683 | 0.014 |
|  |  | GBLUP | 0.301 | 0.028 | 0.681 | 0.014 |
|  | Body Weight | BayesC | 0.486 | 0.058 | ---- | ---- |
|  |  | GBLUP | 0.483 | 0.056 | ---- | ---- |
| M2 | NA | BayesC | 0.376 | 0.066 | 0.700 | 0.040 |
|  |  | GBLUP | 0.379 | 0.063 | 0.699 | 0.043 |
|  | RCCL | BayesC | 0.017 | 0.113 | 0.626 | 0.052 |
|  |  | GBLUP | 0.024 | 0.108 | 0.623 | 0.059 |
|  | Body Weight | BayesC | 0.095 | 0.094 | ---- | ---- |
|  |  | GBLUP | 0.104 | 0.088 | ---- | ---- |
| M3 | NA | BayesC | 0.185 | 0.031 | 0.635 | 0.017 |
|  |  | GBLUP | 0.179 | 0.030 | 0.630 | 0.018 |
|  | RCCL | BayesC | 0.402 | 0.023 | 0.742 | 0.013 |
|  |  | GBLUP | 0.411 | 0.023 | 0.748 | 0.014 |
|  | Body Weight | BayesC | 0.190 | 0.041 | ---- | ---- |
|  |  | GBLUP | 0.193 | 0.039 | ---- | ---- |
| M4 | NA | BayesC | 0.126 | 0.027 | 0.588 | 0.022 |
|  |  | GBLUP | 0.141 | 0.027 | 0.584 | 0.022 |
|  | RCCL | BayesC | 0.373 | 0.025 | 0.723 | 0.017 |
|  |  | GBLUP | 0.367 | 0.022 | 0.718 | 0.015 |
|  | Body Weight | BayesC | 0.184 | 0.032 | ---- | ---- |
|  |  | GBLUP | 0.182 | 0.033 | ---- | ---- |

Table S5. The cross-validation results, using BayesC and GBLUP model, based on 54,858 associated SNPs from imputed genotype data for canine hip dysplasia (CHD), rupture of the cranial cruciate ligament (RCCL), and body weight. NA = Norberg angle, AUC=Area Under the Receiver Operator Characteristic Curve. M1 = random sampling in multi-breed population, M2 = random sampling in Golden Retriever dogs, M3 = random sampling in Labrador Retriever dogs, M4 = random sampling in Labrador Retriever dogs and adding dogs from other breeds in the reference population.

|  | Trait | Model | Pearson Correlation | | AUC | |
| --- | --- | --- | --- | --- | --- | --- |
|  |  |  | Average | SD | Average | SD |
| M1 | NA | BayesC | 0.303 | 0.016 | 0.683 | 0.008 |
|  |  | GBLUP | 0.301 | 0.016 | 0.682 | 0.008 |
|  | RCCL | BayesC | 0.267 | 0.034 | 0.660 | 0.013 |
|  |  | GBLUP | 0.270 | 0.031 | 0.661 | 0.014 |
|  | Body Weight | BayesC | 0.492 | 0.059 | ---- | ---- |
|  |  | GBLUP | 0.493 | 0.056 | ---- | ---- |
| M2 | NA | BayesC | 0.161 | 0.064 | 0.628 | 0.045 |
|  |  | GBLUP | 0.144 | 0.064 | 0.627 | 0.044 |
|  | RCCL | BayesC | 0.136 | 0.092 | 0.643 | 0.052 |
|  |  | GBLUP | 0.110 | 0.096 | 0.636 | 0.054 |
|  | Body Weight | BayesC | -0.120 | 0.108 | ---- | ---- |
|  |  | GBLUP | 0.012 | 0.093 | ---- | ---- |
| M3 | NA | BayesC | 0.250 | 0.034 | 0.659 | 0.020 |
|  |  | GBLUP | 0.247 | 0.035 | 0.657 | 0.021 |
|  | RCCL | BayesC | 0.335 | 0.028 | 0.693 | 0.018 |
|  |  | GBLUP | 0.335 | 0.029 | 0.694 | 0.017 |
|  | Body Weight | BayesC | 0.243 | 0.042 | ---- | ---- |
|  |  | GBLUP | 0.239 | 0.039 | ---- | ---- |
| M4 | NA | BayesC | 0.263 | 0.027 | 0.643 | 0.019 |
|  |  | GBLUP | 0.261 | 0.025 | 0.642 | 0.019 |
|  | RCCL | BayesC | 0.318 | 0.026 | 0.686 | 0.017 |
|  |  | GBLUP | 0.319 | 0.029 | 0.687 | 0.018 |
|  | Body Weight | BayesC | 0.240 | 0.040 | ---- | ---- |
|  |  | GBLUP | 0.241 | 0.037 | ---- | ---- |

Table S6. The cross-validation results, using BayesC and GBLUP model, based on 54,858 randomly selected SNPs from imputed genotype data for canine hip dysplasia (CHD), rupture of the cranial cruciate ligament (RCCL), and body weight. NA = Norberg angle, AUC=Area Under the Receiver Operator Characteristic Curve. M1 = random sampling in multi-breed population, M2 = random sampling in Golden Retriever dogs, M3 = random sampling in Labrador Retriever dogs, M4 = random sampling in Labrador Retriever dogs and adding dogs from other breeds in the reference population.

|  | Trait | Model | Pearson Correlation | | AUC | |
| --- | --- | --- | --- | --- | --- | --- |
|  |  |  | Average | SD | Average | SD |
| M1 | NA | BayesC | 0.337 | 0.014 | 0.699 | 0.007 |
|  |  | GBLUP | 0.360 | 0.019 | 0.703 | 0.010 |
|  | RCCL | BayesC | 0.342 | 0.037 | 0.708 | 0.012 |
|  |  | GBLUP | 0.370 | 0.030 | 0.720 | 0.010 |
|  | Body Weight | BayesC | 0.509 | 0.059 | ---- | ---- |
|  |  | GBLUP | 0.503 | 0.062 | ---- | ---- |
| M2 | NA | BayesC | 0.311 | 0.059 | 0.680 | 0.042 |
|  |  | GBLUP | 0.312 | 0.055 | 0.684 | 0.042 |
|  | RCCL | BayesC | -0.108 | 0.106 | 0.632 | 0.046 |
|  |  | GBLUP | -0.126 | 0.105 | 0.629 | 0.052 |
|  | Body Weight | BayesC | 0.122 | 0.106 | ---- | ---- |
|  |  | GBLUP | 0.161 | 0.093 | ---- | ---- |
| M3 | NA | BayesC | 0.236 | 0.030 | 0.690 | 0.018 |
|  |  | GBLUP | 0.241 | 0.031 | 0.686 | 0.019 |
|  | RCCL | BayesC | 0.454 | 0.020 | 0.776 | 0.013 |
|  |  | GBLUP | 0.464 | 0.020 | 0.779 | 0.014 |
|  | Body Weight | BayesC | 0.289 | 0.034 | ---- | ---- |
|  |  | GBLUP | 0.295 | 0.037 | ---- | ---- |
| M4 | NA | BayesC | 0.274 | 0.025 | 0.689 | 0.017 |
|  |  | GBLUP | 0.282 | 0.033 | 0.672 | 0.020 |
|  | RCCL | BayesC | 0.445 | 0.020 | 0.772 | 0.014 |
|  |  | GBLUP | 0.458 | 0.022 | 0.778 | 0.014 |
|  | Body Weight | BayesC | 0.300 | 0.031 | ---- | ---- |
|  |  | GBLUP | 0.305 | 0.033 | ---- | ---- |

Table S7. The cross-validation results based on imputed genotype data for canine hip dysplasia (CHD), rupture of the cranial cruciate ligament (RCCL), and body weight using BayesC and GBLUP model. NA = Norberg angle, AUC=Area Under the Receiver Operator Characteristic Curve. IMP1 = random sampling in multi-breed population, IMP2 = random sampling in Labrador Retriever dogs with fixed effect (sex), IMP3 = random sampling in Labrador Retriever dogs with dogs from other breeds in the reference population.

|  | Trait | Model | Pearson Correlation | | AUC | |
| --- | --- | --- | --- | --- | --- | --- |
|  |  |  | Average | SD | Average | SD |
| IMP1 | NA | BayesC | ---- | ---- | ---- | ---- |
|  |  | GBLUP | 0.366 | 0.017 | 0.706 | 0.010 |
|  | RCCL | BayesC | ---- | ---- | ---- | ---- |
|  |  | GBLUP | 0.366 | 0.028 | 0.714 | 0.012 |
|  | Body Weight | BayesC | ---- | ---- | ---- | ---- |
|  |  | GBLUP | 0.521 | 0.053 | ---- | ---- |
| IMP2 | NA | BayesC | ---- | ---- | ---- | ---- |
|  |  | GBLUP | 0.262 | 0.030 | 0.697 | 0.018 |
|  | RCCL | BayesC | ---- | ---- | ---- | ---- |
|  |  | GBLUP | 0.545 | 0.017 | 0.809 | 0.015 |
| IMP3 | NA | BayesC | ---- | ---- | ---- | ---- |
|  |  | GBLUP | 0.318 | 0.031 | 0.720 | 0.017 |
|  | RCCL | BayesC | ---- | ---- | ---- | ---- |
|  |  | GBLUP | 0.448 | 0.017 | 0.771 | 0.013 |
|  | Body Weight | BayesC | ---- | ---- | ---- | ---- |
|  |  | GBLUP | 0.233 | 0.039 | ---- | ---- |

SUPPLEMENTAL FIGURES

Figure S1. Venn plot for the number of dogs in canine hip dysplasia (CHD), rupture of the cranial cruciate ligament (RCCL) and body weight.


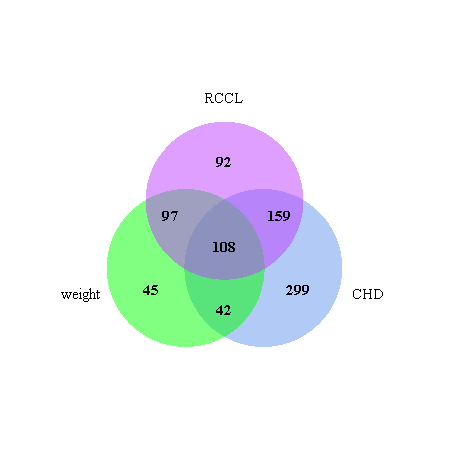

Supplement: Supplementary file 2 [file DataSheet1.docx]
